# Identifying the priorities for supervision by lived experience researchers: a Q sort study

**DOI:** 10.1186/s40900-024-00596-w

**Published:** 2024-06-25

**Authors:** Veenu Gupta, Catrin Eames, Alison Bryant, Beth Greenhill, Laura Golding, Jennifer Day, Peter Fisher

**Affiliations:** 1 Department of Primary Care and Mental Health, University of Liverpool, Liverpool, UK; 2 Department of Psychology, University of Durham, Durham, UK; 3 School of Psychology, Liverpool John Moores University, Liverpool, UK; 4 School of Health Sciences, University of Manchester, Manchester, UK

**Keywords:** Lived experience researcher, Supervision, Q methodology, Reflexivity, Identity

## Abstract

**Background:**

Lived experience researchers draw on their lived and living experiences to either lead on or inform research. Their personal experiences are relevant to the research topic and so they must manage the interplay of their health and healthcare experiences with the research, population, and data they work with, as well as the more general challenges of being a researcher. Lived experience researchers must navigate these dilemmas in addition to queries over their competency, due to issues relating to intersectionality and epistemic injustice. This justifies a motivation to better understand the experiences of lived experience researchers and develop appropriate and personalised supervision based on their preferences and needs.

**Methods:**

Q methodology was used to identify a collection of identity-related issues that impact lived experience researchers during PhD research in the context of the UK. These issues were presented in the form of 54 statements to 18 lived experience researchers to prioritise as topics to explore in supervision.

**Result:**

It was found that lived experiences researchers could be grouped into three distinct factors following an inverted factor analysis: Factor 1: Strengthening my identity, skills, growth, and empowerment; Factor 2: Exploring the emotional and relational link I have with the research and Factor 3: Navigating my lived and professional experiences practically and emotionally. The findings suggest that there may be three types of lived experience researchers, each with different needs from supervision, suggesting the population is heterogeneous.

**Conclusion:**

The research identified a deeper understanding of the needs of lived experience researchers and highlights the importance of personalised supervision according to the individual needs of the researcher and their preferences for supervision. The findings reinforce the importance of integrating a clinical dimension into supervision to support the needs of all lived experience researchers.

**Supplementary Information:**

The online version contains supplementary material available at 10.1186/s40900-024-00596-w.

## Abstract (plain language summary)

This research aimed to understand how to support lived experience researchers through supervision in the UK. Lived experience researchers lead and/or inform health research based on their personal experience of health and healthcare services which can lead to service provision that meets service user needs. People in these roles might be called lived experience researchers. Lived experience researchers may experience difficulties in their role as they conduct research linked to their own experiences. The research could trigger them to remember their own experiences. Other researchers might not value their knowledge and expertise as they are seen as patients and not professionals. This may be because they are viewed through their stigmatised patient identity. The support they need as lived experience researchers remains relatively unexplored. Fifty-four statements on the lived experience researcher experience were presented to 18 lived experience researchers. They were asked to prioritise these statements and arrange them on a grid according to how useful they were to them to explore in supervision. Lived experience researchers who prioritised statements similarly were grouped together. Three groups of lived experience researchers were found. The 1st group were interested in supervision that helped them develop their confidence, growth and professional skills. The 2nd group wanted to understand their personal link to the subject. The 3rd group wanted support to manage personal and professional challenges and to overcome barriers they experienced. The findings suggest different approaches are needed to personalise supervision for lived experience researchers.

**Supplementary Information:**

The online version contains supplementary material available at 10.1186/s40900-024-00596-w.

*Knowing that you can relate to the research topic and the participants because you know you have shared similar experiences is one thing - but knowing how to use this relatability during your work, I find, is a skill I don’t know how to use naturally.*

~ Lived experience researcher.

## Background

Lived experience researchers conduct research on topics related but not limited to, their experiences of mental or physical disabilities, services, and treatments they have experienced, or populations or roles they belong to. Lived experience researchers often draw on their own experiences to lead or inform the design, conduct, analysis, and dissemination of research. It is widely understood that their perspectives will identify different priorities in research compared to that of researchers in the team who do not have lived experience [1]. Lived experience researchers identify and contribute to meaningful research that better meets the needs of the population [2]. As a result of this, the National Institute for Health Research NIHR [3], mandates lived experience involvement in health research.

Lived experience researchers might alternatively be referred to as peer researchers, service user researchers, consumer researchers, user-led researchers, survivor researchers, and disability researchers. Several UK based research organisations support lived experience research, including for example, the NIHR through Applied Research Collaboration (ARC), National Survivor User Network (NSUN), McPin Foundation, and Survivor Researcher Network (SRN), to name a few. Higher Education Institutes also engage in lived experience research such as the Institute of Psychiatry, Psychology and Neuroscience at Kings College London, and University of Birmingham amongst others. Within these settings, those conducting health research employ patient and public involvement (PPI) contributors and/or have dedicated PPI groups informing their research to ensure that lived experience perspectives remain central to the research process. Additionally, there may be lived experience researchers that lead on research in these contexts. The value of these contributions is identified by many experts including PPI contributors themselves [4]. Its importance is also being recognised internationally [5]. The relevance of the lived experience on the research topic or population being studied is additionally gaining importance. In response to this, the NIHR [6] identifies flexible guidance on how to determine what experts to include in research. However, to date, there is surprisingly limited research on understanding the impact of researching areas related to one’s own personal experiences that may for instance be burdensome, triggering and emotionally laboursome as found in research on peer researchers by Faulkner and Thompson [7]. There is little known about lived experience researchers’ support needs.

The lived experience researcher typically spans patient and professional roles that have historically been considered binary [8]. This dichotomy between the patient and professional role may contribute to the many challenges and conflicts the lived experience researcher experiences in health research. Consistent with this, Gupta et al. [9] found, through a systematic narrative review of the literature, that those in lived experience researcher and provider roles, including experts by experience, peer workers, lived experience researchers and mental health professionals with lived experience moved between and navigated multiple personal and professional identities and this affected how they were viewed and which influenced their experiences of exclusion, stigma, and discrimination. The disability researcher, for example, is often perceived in different ways by the people they encounter in different contexts they occupy that does not easily integrate into one disability researcher’s identity. In clinical settings, for example, they may be viewed as disabled people and consumers of services, that may for instance limit the control they have over decisions regarding their healthcare. Whereas in research settings they may be perceived as experts by experience, providing a service with more power and autonomy to shape and positively influence research, service provision and policy [10].

Lived experience researchers may experience additional difficulties based on the personal and social identities they belong to. This may compound the discrimination they experience due to intersectionality [11]. The authenticity and value of their expertise might be queried due to epistemic injustice, which is a term that describes doubting the individual’s knowledge they produce, or their interpretations of knowledge. [12]. Applying this to the lived experience researcher might lead to their knowledge, and sense making of data to be questioned by others as they may be viewed through the lens of their stigmatised patient identity consequently overshadowing their expertise [12]. Epistemic injustice might additionally extend to queries regarding their impartiality to their research, and issues such as bias, and the idea of a credibility deficit might be raised [13].

Reflexive approaches may be of value in lived experience research. This is where researchers actively interrogate their own biases and subjectivity to help them better interpret their research findings which is critical in qualitative research [14]. More specifically, Olmos-Vega et al. [15] highlight how reflexivity is a mark of rigour in qualitative work. Its importance is also being recognised in quantitative research [16]. Watharow and Wayland [17] have applied reflexive approaches to disability research and find that being reflexive makes the participant’s experiences better understood. It additionally makes clearer the accessibility needs and reasonable adjustments disability researchers require when conducting their research. Reflexive supervision could support lived experience researchers to explore their relation to the research topic from the different positionalities of the researcher, participant, and service user [17]. Similarly, Proctor and Winter [18] outline how reflexive supervision can help explore personal, social, and relational views in supervision. Lazard and McAvoy [19] highlight the importance of reflexivity in addressing identity-related issues and the positionality of the researcher. This exploration of identity-related issues and reflexive approaches are likely to support lived experience researchers to effectively conduct their research.

Despite many challenges for lived experience researchers, there are identified benefits. Gupta et al. [9] found that when lived experience researcher and providers integrated their lived experiences into their work led to empowerment, recovery, and growth, moving them beyond the stigmatised service user identity. Other research finds how integration of lived experience perspectives benefits research and those performing these roles [20].

Faulkner & Thompson [7] identify practical ways to support lived experience researchers and consider the value of supervision or peer support. Currently, supervision is conceptualised in two domains (i) clinical, incorporating formative, restorative, and normative support [21], and (ii) academic to support students, early career researchers and those at more senior levels to develop their research skills [22]. Additionally, trauma-informed approaches to supervision have been created [23] including those that incorporate explorations of identity and intersectionality [24]. However, there is no guidance on conducting supervision informed by identity-related issues specific to the lived experience researcher. Currently, clinical supervision is limited to healthcare professionals [25]. However, drawing on and integrating elements of clinical supervision and reflexive approaches into academic research supervision may be appropriate for supporting the needs of lived experience researchers. More guidance is needed on how supervision can be appropriately implemented.

This study aimed to explore the priorities and needs of lived experience researchers from supervision using Q methodology [26]. The study identified statements related to the lived experience researcher experience based on findings from the work of Gupta et al. [9] including from the systematic narrative review (The EMERGES framework: Empowerment; Motivation to Integrate; Empathy of the self and others; Recovery model and medical model; Growth and transformation; Exclusion and Survivor roots) and the positions of identity (Professional; Service user; Integrated; Unintegrated and Liminal) that lived experience researchers move between. The study will use Q methodology to explore the complexity of these issues based on lived experience researcher perspectives to support outcomes that can lead to better tailored research supervision. The needs of those in lived experience roles have not historically been prioritised, resulting in limited research in the area. To the authors’ knowledge, this is the first study that aims to understand how to effectively personalise supervision for lived experience researchers using Q sort methodology.

## Methods

### Aims

To understand the priorities of lived experience researchers for supervision using Q methodology.

### Design

Q methodology [26] a mixed methods approach was used to understand subjective viewpoints towards lived experience researchers’ priorities for supervision in a cross-sectional study.

Q methodology is known as a social constructionist approach which means that it taps into the subjective viewpoints of participants that are built from their own social interactions and experiences of the world. Q methodology is based on the idea that people are active participants in the creation of their own knowledge and understanding of the world. This approach seemed to align closely with the aims of this study and made it an ideal method in this context [26].

### Ethics

The University of Liverpool granted ethical approval on 01/12/2021(Ref 10138).

### Reflexivity and Positionality

VG’s personal experience of being a lived experience researcher motivated the conceptualisation of the research. VG collected this empirical data and carried out the analyses. CE is a research academic psychologist, and clinical academic psychologists, PF, LG, and BG brought their supervisory expertise to the research process and contributed to the conceptualisation and design of the study. CE helped identify the research methodology. Methodology advisor, JD, advised on how to effectively use Q sort methodology for this research question. AB’s lived experience perspectives, as service user advisor, were sought to help identify and clarify statements regarding the supervisory needs of lived experience researchers, alongside the research team and methodology advisor. Each author helped to interpret the findings. Discussions between team members often focused on identifying and differentiating between issues that were specifically relevant to the lived experience researcher experience, issues related to the general experience of researchers and issues that were identity-related. The experiences of members within the research team with lived experience (VG and AB) were reflected on against the findings of the research. VG kept a reflective diary across the research process to manage the challenges of lived experience work and to identify when the data either met expectations or revealed different insights. The GRIPP 2 checklist also details the nature of PPI involvement in the study.

### Recruitment process

The P set is the sample of participants included in the study. To be eligible to participate in this study participants had to be lived experience researchers and self-define as working as either a service user researcher, peer researcher, survivor researcher, disability researcher, user-led researcher or lived experience researcher. The study defines this role as an individual that draws on their lived experience of physical or mental disabilities and uses it to inform or lead on and conduct research that is to some degree related to their own lived experiences, either independently, at a university, charity, third sector organisation or in the NHS. Participants were recruited from organisations by purposive sampling via email invitation to take part. Participants had to read and understand the information sheet to provide informed consent prior to participation. If eligible, they were asked whether they wanted to participate either online, in person or via post. Those who decided to participate online were sent the link to participate in the study. Those who took part via post were sent materials to complete the study and a stamped addressed envelope to return their completed materials. These different methods were used as the data was collected during the COVID-19 pandemic. Following completion of the study, participants were provided with the debriefing form detailing the study’s aims, the complaints procedure, contact emails of the research team, and organisations to contact for support if needed. All participants were offered a £10 voucher to value their participation.

### Step 1 item development

The Q set is the statements presented to the participants that represent the breadth of a topic. In this study, the Q set was developed by gathering statements from the lead author’s PhD research specifically about lived experience researchers and providers [27]. Statements were identified under themes and key areas related to the experiences of lived experience researchers. These themes were generated from the systematic review in the EMERGES framework (Empowerment and Enablers, Motivation to integrate, Empathy of the self and others, Recovery model and medical model, Growth and transformation, Exclusion and stigma and discrimination and Survivor and disability experiences) [9], and ideas related to personal and professional identity, reflexivity, experience, and support needs. It was also informed by two empirical studies and reflections of the lived experience researchers in the PhD thesis [27]. Forty-to-eighty statements are thought to be ideal for Q methodology [28]. The Q set statements were reviewed by the supervisory team and service user advisor to ensure clarity and comprehensibility. The statements were revised based on this feedback. To reduce the cognitive demand of the task on the participants, it was thought appropriate to have fewer statements. The research team, therefore, focussed on statements specific to the lived experience researchers as opposed to the general needs of researchers. The Q set was reduced from an initial 80 to 65 statements and then further reduced to 54 by excluding similar statements and making others represent broader themes.

### Step 2 how the Q-sort was conducted

**Fig. 1:**
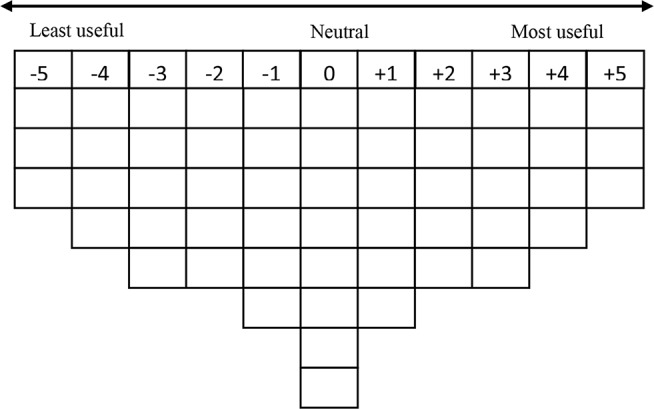
Forced Q sort distribution grid

A pilot study was conducted with five participants who completed the Q-sort. Based on feedback from this pilot the protocol was refined and adjusted to enhance readability and clarity of instructions and statements. Participants were given the option of doing the Q sort task with the researcher present to guide participants through the task either in person or completing the task on an online platform (i.e. using a laptop or computer) or via post (i.e. completing the task in paper format and sending this data to the researcher). A total of 27 people agreed to participate. However, seven people did not complete the task and two had missing data, which left an overall sample size of 18. No participants took part in person, six participants took part online and twelve participants via post. It is recognised that effective Q sort methodology can be done with a sample of less than 20 participants [26]. The instruction given to participants was: *“Please arrange the items related to supervisory topics according to how useful they are to you to discuss in your supervision as a lived experience researcher”*. Participants were first asked to arrange the 54 statements into three categories of most useful, neutral, and least useful to them. They were then asked to use this initial sorting of the statements to support them to arrange these same statements on a pre-defined Q sort grid on an 11-point Likert scale based on the same research instructions using a Likert scale of – 5 least useful to + 5 most useful (see Fig. 1). Participants were able to move these statements around on the grid until they were happy with the way they had ranked them.

The statements were presented in a random order for all participants, and not structured under any themes. There was an acknowledgement that there may be more statements participants find more or less useful than the options available to them on the pre-defined grid. Participants were given the opportunity to discuss their decision-making on how they sorted the statements in the task and to leave further feedback to help contextualise findings (see supplementary materials). Participants were advised to take breaks whilst undertaking the task due to cognitive demand and were reminded that they could withdraw from the study at any point without negative repercussions to them.

### Data analysis

The data was analysed using KenQ software [29] by uploading an Excel document with the data entries in. A principal component analysis was used with a varimax rotation. This statistical process is used to identify people who have sorted the statements in a similar way and to quantify how much each person agrees with (loads on) each Factor. The number of factors extracted was determined based on the factor loadings, scree plot, eigenvalues, and cumulative variance each participant contributed to the factors, and these judgements were based on the expertise of the researchers [30]. The variance is the amount of variability in the data explained by each factor and is usually expressed as a percentage. Eigenvalues are calculated for each factor by multiplying the variance by the number of participants and then dividing this by 100. Factors that have an eigenvalue of more than 1.00 are extracted by the software. Kline, [31] identifies how the bigger the eigenvalue, the greater it explains the variance within the data. Q methodology typically extracts 7 or 8 factors, but it is acknowledged that the latent variables in the data are typically limited to 3 or 4 factors according to Kline, [31].

**Table 1:** Participants

**Elanor **–has had “Positive” experiences of lived experience research and is involved in this work “to aid my recovery.” She has been working in the field for 10 years.	Jenny says, “I feel valued, have a voice and feel a part of the team.” She has been working in the lived experience field for 11 years.
**Kate** – has been working for 5 years in her role “I was most interested in areas in which I had lived experience - both mental distress and LGBTQ+ wellbeing. To me it makes sense and is vital that research in these and other marginalised areas are led by people who’ve experienced what they are investigating.”	**Tom** – has had a “difficult mental health journey” and is involved as a lived experience researcher to “Inform research and policy making.” Has been working in the field for 5 years.
**Rosena** – has been a lived experience researcher for 3 years and is engaged in work on advanced directives because she “wants a voice should I become unwell.”	**Emily** has been working in lived experience for 5 months. She has experienced coercive control and researches restorative healing for survivors of coercive control.
**Alice** - has been working as a lived experience researcher for 3 years. When referring to lived experience work says, “I’d never heard this concept before but thought it was really great that I could use my negative experiences to help improve these experiences for others.”	**Alex** – Works as both a patient and public involvement consultant but now also as a research assistant outside of this field but is still engaged in lived experience work. They have been in the field for 6 years and “Just fell into it! But wanted to help others.”
**Sarah** – has been a lived experience researcher for 5 years. She finds the role rewarding but at times challenging. She has experiences of being racially bullied and researches this experience in children. She says, “Sometimes being so involved with the data can be very difficult but knowing that I am helping others with similar experiences can make this feel worth it/easier.”	**Judy** – has been working as a lived experience researcher for 9 years. She finds that being disabled and having the stigma of a mental health condition is a detraction from your status as a researcher. “It’s a travesty that I’m expected to pretend that everything is ok – even though I’m marginalised because of my race, gender, and the impact of my mental health. I don’t intend to be quiet anymore!”
**Jin** – has been a lived experience researcher for 4 years. He says, “I want to help better services and interactions between clinicians and patients to equalise these roles so there isn’t a power difference.”	**Julie** – finds the lived experience researcher role “Interesting, varied and enjoyable.” She has been in the role for 12 years and says, “I feel connected to the research I do when I am a service user researcher.”
**Jane** – “Generally positive” experiences of being a lived experience researcher.	**Meena**- has been working in lived experience roles for 2 years. She says, “I needed a job, a supportive role and a route into academic research.”
**Rani** – has been working as a lived experience researcher for 9 months and says, “Using my lived experiences has been empowering.”	**Emma** – 4 months into PhD, she says due to her personal connection to the research “I understand that I have personal bias and I need to be objective in my research.”
**Lina** – Says her work as a lived experience researcher was “Accidental! I just happen to research a topic that affects me.”	**Caroline** – has 4 years of experience working in lived experience work. She had poor mental health during her PhD. She wants to “Try and make a contribution so no one, or less people, go through what I did.”

Table 1 presents the pseudo-anonymised participants in the study and their positive and negative experiences of being a lived experience researcher and the length of time in their role.

**Table 2:** Factor arrays and composite loadings

Statement	Factor 1	Factor 2	Factor 3
1. Discussing how to use the skills and abilities I already have and apply them in my role	+ 4	+ 2	-3
2. Identifying my training and learning needs relevant to the research	+ 4	+ 1	− 1
3. Supporting me with issues around payment for my work by my employer	− 1	-5	+ 4
4. Discussing whether my professional experiences impact the research	-4	− 2	− 2
5. Exploring what it means to be a lived experience researcher and enabling me to better understand the role	0	0	+ 3
6. Managing the expectations that I and others have of me in this role	+ 2	-4	+ 5
7. Co-creating appropriate labels/job titles for my role	− 1	-5	-5
8. Supporting me with queries over my fitness to do my role	-5	-5	− 2
9. Having regular opportunities to discuss personal difficulties that may impact my role	+ 5	+ 1	+ 2
10. Discussing queries relating to my competence as a researcher due to my personal connection to the subject	-5	-3	-4
11. Talking about my subjectivity and objectivity in relation to the research area	+ 2	+ 1	-4
12. Enabling a better understanding of the boundaries and remit of my role	0	− 2	+ 1
13. Helping me to set boundaries between my personal life and professional role	-3	0	0
14. Helping me to know when to separate my personal and professional experiences	− 2	− 2	− 1
15. Exploring how I relate to the research topic	0	+ 4	-4
16. Exploring how I relate to the participants in the research	− 2	+ 4	-3
17. Exploring how to navigate lived experience and professional aspects of the work at the same time	− 1	− 2	+ 3
18. Exploring how to actively integrate learning from my lived experience and apply it to the research	+ 1	0	− 2
19. Strengthening my identity as a lived experience researcher	+ 4	− 1	0
20. Helping me to feel part of the team	+ 1	0	+ 3
21. Discussing aspects of myself that are known to others and how this may impact my work	0	0	+ 3
22. Enabling me to disclose aspects of myself that are unknown to others and discussing how this may impact my work	+ 1	− 1	+ 2
23. Helping me to reflect on how I feel when assumptions are made of me due to my lived experience	-3	+ 4	− 1
24. Helping me to increase my confidence in my role as a lived experience researcher	+ 2	− 1	− 1
25. Helping me to feel valued and validated through supervision	+ 5	+ 5	0
26. Helping me to be heard as a lived experience researcher in the team	+ 3	+ 3	+ 5
27. Helping me to identify the positive experiences I have in my role	+ 3	− 2	-3
28. Helping me to identify sources of empowerment in relation to my role	+ 3	-3	+ 1
29. Enabling discussion on experiences of disempowerment in my role	+ 2	0	+ 2
30. Enabling discussion about the emotional burden the role may have on me	+ 1	+ 3	+ 3
31. Discussing the political motivations that underlie my work	-3	-3	0
32. Enabling me to reflect on what motivates and enables me to do my role	+ 4	+ 2	0
33. Providing space to discuss the impact on me when seeing people like me suffer through the data	-3	+ 5	− 1
34. Providing space to discuss the impact on me when seeing people like me recover through the data	-4	+ 2	+ 2
35. Helping me to reflect on how I feel when research findings are similar to my own experiences	-5	+ 2	0
36. Helping me to reflect on how I feel when research findings are different to my own experiences	− 2	+ 2	− 2
37. Providing space to discuss the social groups I am a part of and whether this impacts the research	-4	-4	0
38. Providing space to discuss the social relationships I have within the team I work in	0	-4	-5
39. Providing opportunities to share and learn through others experiences within the research	+ 1	+ 3	0
40. Reflecting on working with individuals that understand their experiences differently to me	− 1	− 1	-3
41. Reflecting on working with individuals that understand their experiences similarly to me	-4	0	-4
42. Reflecting on power differences I experience in my role with those I work with	+ 1	-3	-5
43. Having conversations in which there is recognition of my growth in my role	+ 3	− 1	− 2
44. Identifying when I am making a difference and to be praised and acknowledged for this	+ 5	+ 1	+ 5
45. Discussing the positive or negative impact I am having on the research	0	+ 1	-3
46. Enabling me to share my experiences of exclusion	− 1	− 4	− 1
47. Helping me to reflect on times when I am prevented from making meaningful change	− 1	0	+ 4
48. Helping me to reflect on barriers I come across in my role	0	+ 1	+ 4
49. Helping me to reflect on the stigma or discrimination that I personally experience	− 2	-3	+ 1
50. Helping me to reflect on the impact of witnessing others experience stigma or discrimination	-3	− 1	+ 1
51. Discussing additional support or reasonable adjustments I require to do my role	0	+ 3	+ 2
52. Providing space for me to share and reflect on my previous history of lived experience	− 2	+ 3	+ 4
53. Providing space for me to talk about my current lived experiences and how this may impact the research	+ 3	+ 5	+ 1
54. Regularly discussing and reviewing my wellbeing and support needs	+ 2	+ 4	+ 1

Table 2 shows the position of each statement for each composite factor array. For example, people who loaded on Factor 1 strongly agreed with statement 1 (+ 4), people who loaded on Factor 2 weakly agreed with statement 1 (+ 2), and people who loaded on Factor 3 disagreed with this statement (-3).

**Table 3:** Principal components analysis with varimax rotation

Component loadings for each factor				
Outcome Priority Factor	Participant	Factor 1	Factor 2	Factor 3
**Factor 1** **Strengthening my identity, skills, growth, and empowerment**	Rosena	**0.7623**	0.1616	0.058
	Rani	**0.7318**	0.0846	0.063
	Kate	**0.6504**	0.2638	-0.2198
	Jenny	**0.632**	0.1105	0.0748
	Meena	**0.5808**	-0.3111	0.3674
	Julie	**0.4575**	-0.0448	-0.0318
	Emma	**0.3605**	0.163	0.2772
**Factor 2** **Exploring the emotional and relational link I have with the research**	Sarah	-0.1633	**0.6744**	-0.2315
	Caroline	0.0569	**0.6735**	0.2336
	Alice	0.4122	**0.63**	0.1089
	Emily	0.3874	**0.6179**	0.0728
	Lina	-0.0605	**0.5496**	0.4918
	Judy	0.0889	**0.4385**	0.0176
**Factor 3** **Navigating my lived and professional experiences practically and emotionally**	Elanor	0.2396	0.0048	**-0.6139**
	Alex	-0.3423	0.2793	**-0.5895**
	Tom	0.0102	0.0009	**0.454**
	Jane	0.0404	0.1865	**0.4249**
	Jin	0.2773	0.1329	**0.4051**
**Eigenvalues**		3.84	2.15	1.69
**Variance explained** (of rotated factors)		18%	14%	11%

Table 3 shows the loading of the participants on each factor and there are clear differences in the loadings between the different factors. The factor loading value varies from − 1 (negatively endorsed i.e. disagree with Factor) to 0 (neutral) to + 1 (positively endorsed, i.e. agree with Factor) which are the correlational loadings participants have to each factor. For example, Rosena loads strongly on Factor 1 (0.7623) but only very weakly on Factor 2 (0.1616) and Factor 3 (0.058).

**Table 4:** Demographics of participants loading on each factor

Factors	Demographics (*N* = 18)
Factor 1: Strengthening my identity, skills, growth, and empowerment.	7 Participants. All Female. Age range of 23 years old to 61 years old. 1 Asian Bangladeshi, 1 Indian, 3 White British, 1 Mixed Asian/White, and 1 White other. The range of duration in lived experience work spanned 4 months to 12 years.
Factor 2: Exploring the emotional and relational link I have with the research.	6 Participants. All Female. Age range of 24 years old to 61 years old. 1 participant Mixed White British and Black, 1 White Irish, 1 White, 1 White British, 1 White European and 1 African Caribbean. The range of duration in lived experience work spanned 5 months to 9 years.
Factor 3: Navigating my lived and professional experiences practically and emotionally.	5 participants. 3 female and 1 male and 1 did not disclose. Age range of 34 years old to 50 years old, 2 White British, 1 White Scottish, 1 White European, and 1 Chinese. The range of duration in lived experience work spanned 4 years to 10 years.
	Lived experience (not linked to demographics to ensure confidentiality) Autistic spectrum disorder, Depression, Schizophrenia, Bipolar disorder, Complex PTSD, General Anxiety disorder, Body dysmorphic disorder, Dissociative identity disorder, Self-harm, Suicide attempt, Ankylosing Spondylitis, Fibromyalgia, Obsessive compulsive disorder, Chronic pain syndrome, Pervasive developmental disorder, Disordered eating, Personality disorder, Trauma, Burnout, Voice box paralysis

Table 4 presents the demographics of participants that loaded onto the three factors, their ethnicity, gender, age, and disability.

## Results

**Fig. 2:**
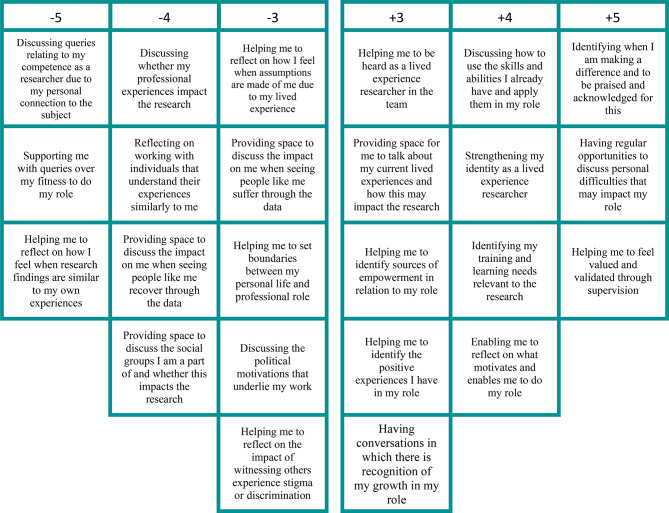
Factor 1: Strengthening my identity, skills, growth, and empowerment

**Fig. 3:**
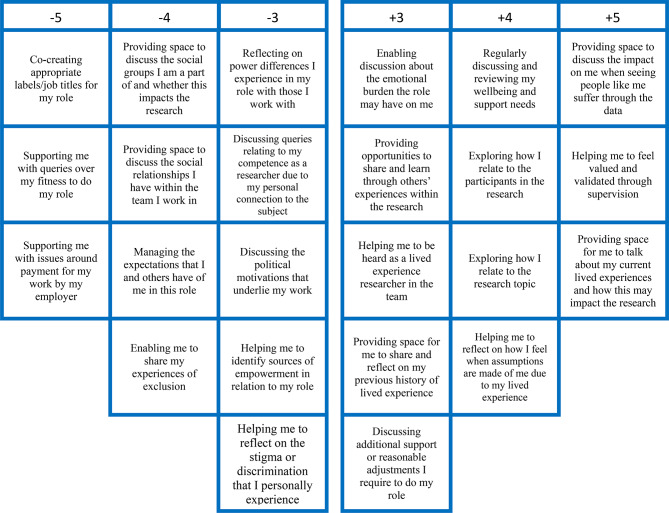
Factor 2: Exploring the emotional and relational link I have with the research

**Fig. 4:**
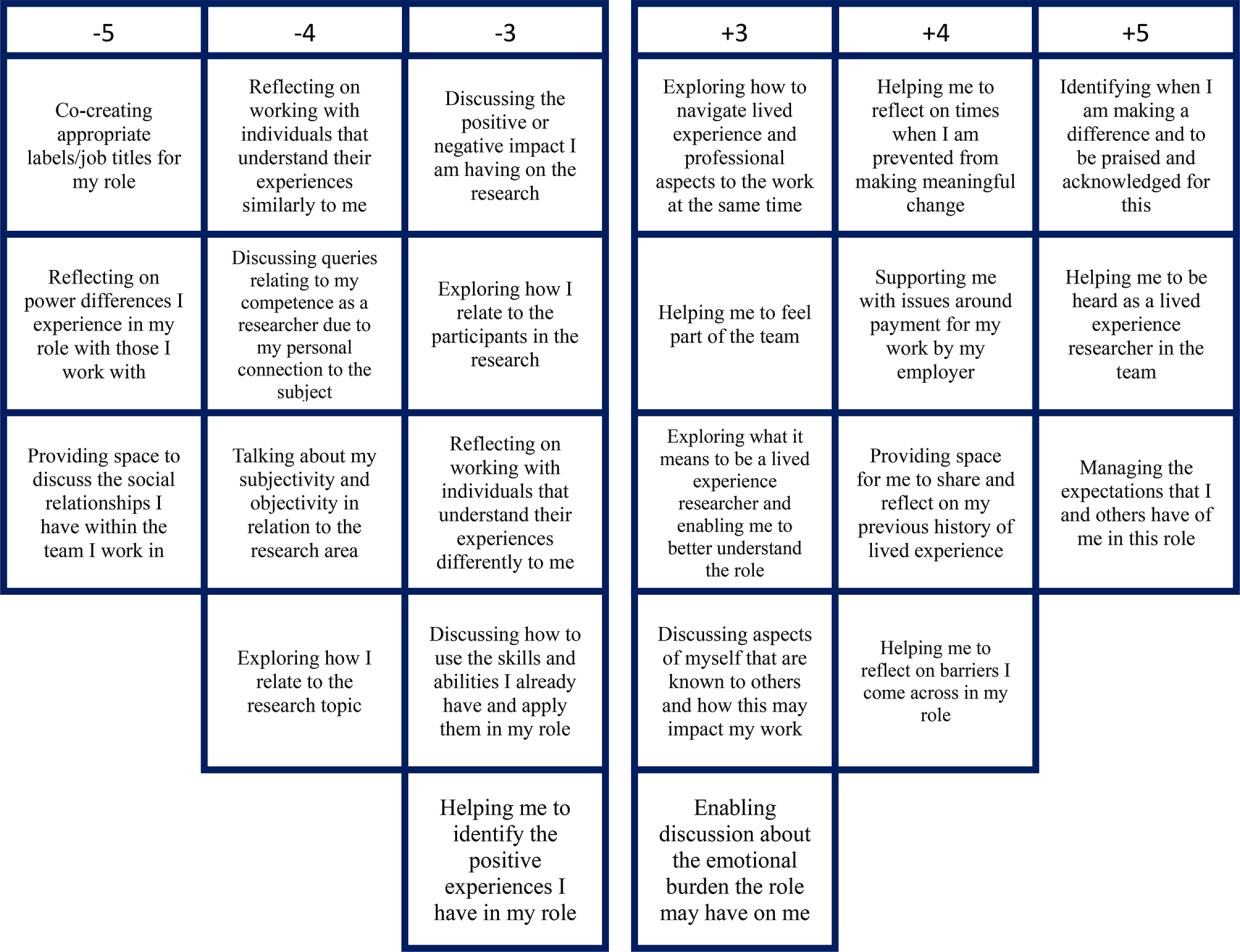
Factor 3: Navigating my lived and professional experiences practically and emotionally

The KenQ analysis initially forced the data into eight factors. Following a varimax rotation in a principal components analysis and a judgement of factor loadings, scree plot, eigenvalues, and cumulative variance, and based on the expertise of the research team [30], three groups of factors were identified of lived experience researchers with different priorities from supervision. The statements from each pole of each factor are presented in Figs. 2, 3 and 4 and distinguishing and significant statements are presented in supplementary Tables S5, S6 and S7) which can help interpret the factors. Factor 1 was labelled Strengthening my identity, skills, growth, and empowerment and 7 participants loaded significantly (all positively) on this factor. Factor 2 was labelled Exploring the emotional and relational link I have with the research and 6 participants loaded significantly (all positively) on this factor. Factor 3 was labelled Navigating my lived and professional experiences practically and emotionally and 5 participants loaded significantly (3 positively and 2 negatively) on this factor. There were clear loadings for each participant across the factors extracted. The factors were independently labelled by the PhD researcher, methodology advisor and supervisory team and there was good agreement between them. These factors are presented next with contextual qualitative feedback from the pseudonymised participants. Service user advisor, AB also helped interpret and contextualise findings in discussion sessions with lead researcher, VG, who also has lived experience to better explore and understand the findings.

### Factor 1: strengthening my identity, skills, growth, and empowerment

This first factor array of lived experience researchers was the most prominent factor array with an eigenvalue of 3.84 and explained 18% of the variance in the data after factor rotation with 7 participants loading positively onto this factor. The factor described lived experience researchers’ needs for supervision to strengthen their identity, with motivations to enable their development through skills and training, and a need for supervisors to recognise their growth and confidence (see Fig. 2; Table S5 supplementary materials). This factor represented more individuals from ethnic minority backgrounds and those who experienced severe mental illnesses including Schizophrenia and Bipolar disorder, which may have contributed to the manifestation of this factor centred around growth and empowerment.

Kate, said, “*identifying my training and learning needs, helping me to feel valued and validated & having regular opportunities to discuss my personal difficulties were the answers that most match what I consider professional non-clinical supervision to be for…I’d expect in any supervision…*.” highlighting how skills development should be the standard expected in research supervision for the lived experience researcher.

Meena said, “*I think empowerment and recognition of growth are more of a priority*,” identifying the importance of this factor.

In contrast, items that were negatively endorsed on this factor were centred around discussing experiences that were disempowering and that alluded to a sense of inadequacy that participants’ lived experiences might bring to the role. Participants in this factor were not concerned with relational experiences or exploring similarities between themselves and the participants in their research (see Fig. 2). The item, “providing space for me to share and reflect on my previous history of lived experience,” was rated lower in this factor in comparison to Factors 2 and 3. This could be interpreted as those who fit under this category do not want to discuss their past experiences as it might detract from a focus on their growth and progression as lived experience researchers and their distance from their service user experiences.

### Factor 2: exploring the emotional and relational link I have with the research

This second factor array had an eigenvalue of 2.157, adding 14% to the variance with a cumulative variance of 32% after factor rotation. Six participants loaded onto this factor. This second factor array was related to ideas of reflexivity and the need for this lived experience researcher to explore their personal connection to the research and participants in the data, the emotional burden of the role, and their well-being needs (see Fig. 3; Table S6). The participants that loaded onto this factor had multimorbidity and some with experiences of mental illnesses such as Body Dysmorphic disorder and disordered eating, which might be considered conditions focussed on relational experiences, potentially resulting in the manifestation of this relationally centred factor. The participants in this factor were engaged in work that was directly linked to their own lived experiences.

Alice, who loaded onto this factor said, “*Knowing that you can relate to the research topic and the participants because you know you have shared similar experiences is one thing - but knowing how to use this relatability during your work I find is a skill I don’t know how to use naturally.*” Alice identified the need to be supported to develop skills to effectively integrate her lived experiences in the research process more meaningfully.

Sarah explained how she wanted support to understand her relation to the data and its impact on her, “*Providing space to discuss the impact on me when seeing people like me suffer through the data - can be quite triggering to hear about/read about participant’s experiences. I think it’s really important to talk to supervisors on a personal level (peer to peer), exploring how I relate to the research topic - It’s really important to understand how my own experiences may impact/influence my interpretations of participant data, and to reflect on this if necessary*.”

Participants that loaded onto this factor were least concerned with items related to social relationships (see Fig. 3) which may mean this lived experience researcher was more focused on their individual relation to the research as opposed to their needs for social connection. “Enabling me to share my experiences of exclusion, − 4.” was negatively loaded on in this factor in contrast to Factors 1 and 3. This might be because experiences of exclusion actively influence the separation of lived experiences from professional roles due to stigma that can discourage health-seeking behaviours or disclosures of lived experiences. Whereas this factor is about effectively integrating lived experiences into their research.

### Factor 3: navigating my lived and professional experiences practically and emotionally

This third factor array had an eigenvalue of 1.689 and added 11% variance resulting in the factors explaining a cumulative variance of 43% after factor rotation. Five participants loaded onto this factor: three positively and two negatively. This factor array was related to exploring and navigating lived and professional aspects of the lived experience researcher role with a desire to be supported both in practical and emotional ways to deal with barriers they experience in their roles (see Fig. 4; Table S7 supplementary materials). Most participants that loaded onto this factor were white, and so may not have experienced the same types of intersectional exclusion as those from ethnic minorities.

Participants identified how they wanted support to carry out their roles and enable the negotiation of both personal and professional experiences. Rosena, who did not load onto this factor but identified its importance, said, “*I do not want too much of my personal lived experience to get in the way of being able to do the role… I have a personal life, but still need support to actually do the role in practical terms, yet with my supervisor providing emotional support when needed*.” This suggested she wanted personalised support based on her needs.

Jin identified how payment was an additional supportive factor enabling him to perform his role, “*Recognition in pay because that is how I will be able to “escape” the mentalhealth system, to leave the benefit system and be free… to do this, I need support and help as I continue to suffer from my condition*.”

Elanor and Alex, loaded negatively onto this factor and each had been working in lived experience work for a significant number of years, having had positive experiences, which might have meant they had gained a level of expertise in their roles and were not concerned with practical or emotional support from supervisors.

People who loaded on Factor 3 tended to negatively endorse items that related to low self-efficacy as a researcher due to lived experiences (see Fig. 4). As shown in the factor arrays, the item, “Discussing how to use the skills and abilities I already have and apply them in my role” was rated lower (-3) in comparison to Factors 1 (+ 4) and 2 (+ 2). This might have been due to a belief that their existing skills may not be sufficient in being able to manage any barriers they experience, necessitating the need for tailored supervision. Elanor and Alex, in contrast, prioritised this which may have been due to their level of expertise in their roles, which may have meant they wanted guidance to draw on their existing skills, see Table 1 for more details on participants. Figure 4 illustrates the spectrum of needs across this factor which reinforces the holistic needs of the lived experience researcher, both personally and professionally.

### Additional topics to explore in supervision

Participants were asked about additional important topics to consider in supervision, adding further nuance to the findings. These suggestions were grouped into three themes that directly mapped onto the findings of the main data: (1) Academic and methodological knowledge of supervisors, (2) Relational experiences and (3) Providing practical and emotional support.

### Academic and methodological knowledge of supervisors

Rani and Kate identified the value of supervisors who could advise on multiple methodologies. Kate stated, “*It would also have been useful to have had a supervisor who is more aware of methodological issues, as it would have been great to explore and frame our research within specifically anti-oppressive or decolonising approaches*.” Caroline, who is also a supervisor explained that what, “*a student struggles with was writing academically rather than emotionally and justifying arguments based on literature rather than personal experience*.” These examples demonstrate how supervisors bring their own expertise to lived experience research supervision and understand the development needs of lived experience researchers. This theme aligns with Factor 1, in terms of skills development.

### Relational experiences

Tom identified that supervisory support should include exploring the impact of, “*power relations within institutions*,” referring to senior faculty, colleagues, and research team members by the lived experience researcher. Emma said “*feeling welcome in the workplace*” was important. Jane additionally stated the importance of learning skills as an academic in, “*managing conflict or difficult relationships*.” Sarah felt that supervisors should do more to facilitate “*connecting people…and facilitating friendships*.” These examples could be interpreted as the need for lived experience researchers to have a sense of belonging. Kate additionally highlighted the need to talk about vicarious and direct experiences of discrimination, “*One thing I’ve found really useful in my current role is the ability to offload to my supervisor when I do come across discriminatory attitudes or assumptions about people with lived experience*…” This example highlights how lived experience researchers experience a shared sense of injustice with each other. These statements relate to findings from Factor 2 but emphasise more social rather than individual relational needs.

### Providing practical and emotional support

Participants identified a need to be supported emotionally and practically. Three participants, Sarah, Emily, and Judy identified the importance of being signposted to support services. Sarah said it would have been, “*useful for supervisors to signpost PhD students to relevant support services e.g., university counselling. This can enable you to focus more on your actual project/research during supervision meetings without personal life taking over.*” Lina identified the importance of, “*Explicit discussion of what is just researcher experience and when external clinical supervision is needed…clinical supervision is vital arguably, especially for lived experience researchers.*” These ideas highlight the importance of distinguishing between different needs and personalising support, echoing factor 3.

### Comments on factor arrays and additional feedback

Being emotionally validated and/or being heard and understood as a lived experience researchers were commonly scored as prioritised across the factors, suggesting that this should be a fundamental component in supervision for all lived experience researchers. Elanor found the process of the Q sort study “*very meaningful*,” and Emily said, “*I wish the outcome of this research was already implemented! Not many of these statements have been presented in my supervision, thus far*,” suggesting the novel nature of the research and the importance of these discussion points.

## Discussion

This research aimed to understand the needs of lived experience researchers and their priorities for discussion in supervision through Q methodology. The findings of the research discovered lived experience researchers could be grouped into three factors: Factor 1; Strengthening my identity, skills, growth, and empowerment. Factor 2; Exploring the emotional and relational link I have with the research. Factor 3; Navigating my lived and professional experiences practically and emotionally. The factors have been labelled based on the academic and lived experience expertise of the team. The following discussion will explore how these factors fit with the wider literature, and how the supervisory process can support lived experience researchers in this emerging field. Each of the factors identifies the importance of emotional support from supervision.

### Factor 1: strengthening my identity, skills, growth, and empowerment

This factor accounted for the highest proportion of variation in the data and identified a need for skills development of the lived experience researcher so they can effectively carry out their role. This relates to the EMERGES framework by Gupta et al., [9] and the theme of empowerment which is centred around combining existing skills with new learning through training, consequently promoting professional development. Dunlop et al., [32, p10] identify in their sharing lived experience framework that “training, support or professional grounding” is often not provided to those in peer support worker roles, justifying a greater focus on their professional development.

This theme may have manifested as the lived experience researcher is often someone who belongs to multiple intersections. For example, all participants who loaded significantly on this factor were female, had multimorbidity, and some were from ethnic minorities, who were more likely to doubt their professional legitimacy and experience imposter syndrome [33] that may have contributed to this factor focused on growth and skills development. Maxwell, [34] identified how peer support training helped counter the experience of imposter syndrome in peer support workers, which may also be of benefit to lived experience researcchers who fit under this factor. Similarly, Simpson et al., [35] found that peer workers’ professional identities were enhanced through training. Training for the lived experience researcher may better strengthen their understanding of the dual identities of the role they occupy. It may also lead to professionalised identities and move them further away from their service user identity. Additionally, Kirrane et al., (36) found retention of researchers was associated with empowering supervisory relationships. This could also possibly allay the negative impact of lived experiences. Lived experience researchers who loaded on this factor wanted to distance themselves from sources of disempowerment which fits with the assumption they want to move beyond their lived experiences. Therefore, to support this workforce it necessitates supervision that is empowering and helps strengthen identity, aligning with a strengths-based approach to supervision (37).

### Factor 2: exploring the emotional and relational link I have with the research

This factor accounted for the second highest variation in the data and identified the lived experience researcher’s need to explore their lived experiences, subjectivity, and reflexivity in relation to their research. For lived experience researchers who loaded on this factor, it was important to them to understand their impact on the research and, vice versa, the impact of the research on them. They were also more likely to be conducting research that was closely tied to their own personal experiences. The importance of reflexivity is recognised in qualitative [14] and quantitative research [16] and may be useful to the lived experience researcher. Poremski et al., [38] found the process of reflexivity was more important to the peer support worker who was introspective. Discussion and recognition of the emotional labour of the work was also considered essential [7].

Participants are likely to be impacted by interactions between the data and their own lived experiences, as illustrated in an anonymous blog by a peer researcher [39]. This suggests that the lived experience researcher may compare and evaluate the validity of their own distress with the people in the research data and those they work with. Reflexive practice may be particularly important to explore these issues. Peer supervision can also support the learning of the supervisee by understanding their own experiences in relation to the experiences of peers [40]. As there is an interaction between the lived experience researchers’ roles and service user experiences, a more clinical dimension to supervision may be of benefit, such as for example, drawing on psychoanalytic or trauma-informed approaches to supervision [41, 23]. Clinical supervision is mandatory for all healthcare professionals [25] but does not apply to lived experience researchers, although this factor suggests academic research supervision should incorporate elements of clinical supervision to better support the lived experience researcher.

### Factor 3: navigating my lived and professional experiences practically and emotionally

This factor was more difficult to label due to the spectrum of statements prioritised, but the research team agreed that the lived experience researcher in this factor was focused on understanding how to navigate both their lived and professional experiences in their roles through supervision and to overcome barriers through practical and emotional support. This theme parallels the positions of identity found in Gupta et al’s [9] systematic review, where lived experience researchers needed to negotiate and move between service user and professional identities that were sometimes integrated or unintegrated. Lived experience researchers who loaded on this factor required the supervisor to be responsive to their changing needs and identities, fitting with Bernard’s discrimination model of supervision [42] and a person-centred approach [43]. Research suggests that those with mental health or physical disabilities are likely to have additional problems, including financial difficulties [44]. This factor identifies the role of the supervisor in addressing different types of needs by providing holistic support, spanning practical, emotional, and financial support [45].

### How the findings relate to other frameworks

The findings across the study coalesce with Proctor’s extant model of clinical supervision that encompasses formative (developing skills and abilities) restorative (supportive of the burden and relational aspects of clinical work) and normative (administrative and managerial) support [21]. These types of support directly map onto the factors found in this research relating to skills and identity development, exploring relational experiences to the research and practical and emotional support. Emotional validation and/or being heard and understood was important to all lived experience researcher participants and so drawing on the common factors of psychotherapy may additionally have relevance to lived experience researchers in supervision. This includes a therapeutic alliance, empathy, unconditional positive regard, and genuineness [46].

### Strengths and limitations

This is the first study to the authors’ knowledge that aimed to understand the views of lived experience researchers and their supervisory needs. Each of the participants loaded onto one of the three factors, with factor distinctiveness, that can support personalised supervision. The factors were independently labelled by the PhD lived experience researcher, supervisors, and methodology advisor, with good agreement between them, reinforcing the validity of the factors.

The sample consisted of predominantly white females, so the findings are not representative of all lived experience researchers, however, the purpose of Q methodology is to identify subjective viewpoints and not to make inferences about the population. The participants in the research identified the importance of exploring stigma and discrimination in their work and advocated for better equality, diversity, and inclusion in the field.

The empirical study was cross-sectional, measuring the perspectives of lived experience researchers at one time point which means their support needs may differ in the future. The exercise can be repeated between lived experience researchers and supervisors regularly to identify their current and changing needs which can support personalised supervision.

Some participants did not complete the task correctly and did not allocate all the statements onto the grid, which led to missing data and some attrition. This might have been because the instructions may have lacked clarity or there may not have been enough access to guidance from the researcher when taking part online or via post. Due to the research being conducted during the COVID-19 pandemic, it led to more participants wanting to participate remotely. Although it is recognised in-person participation is more effective in guiding participants through the task, as the task is more complex than a questionnaire. The variation in participation across online, and via postal participation may have contributed to different experiences of participation. Although all participants had access to the researcher via online conferencing services, but not all participants took up this offer. Some participants reported they did not like the requirement to allocate each statement to a position on the pre-defined grid, as all statements were considered useful, leading to dissatisfaction with the task. Although this dissatisfaction is common in Q sort methodology. Participants identified how the statements were not typical of their supervision but how they would all be beneficial to them.

The concourse may not have been exhaustive of the issues that lived experience researchers experience. However, the research identified additional themes to explore in supervision that mapped onto the three factors found in the data, reinforcing the validity of the factors.

#### Implications

The statements in the Q set can be used as a tool kit to support tailored supervision for lived experience researchers. The three factors draw on different aspects of the EMERGES framework [9, 27] that can further guide personalised supervision, as illustrated in a blog for the McPin Foundation [47].

The implications of these findings necessitate a hybrid supervision, that spans both clinical and academic components of supervision as there is an emotional dimension to the work that manifests in each lived experience researcher. The research offers an original contribution to the field of lived experience research.

## Conclusions

The research identifies the supervisory needs of lived experience researchers. The findings can support supervisors in providing tailored supervision to them. It was found that lived experience researchers could be grouped into three categories each with different priorities and needs for supervision. They were either interested in developing their skills and growth as a lived experience researcher (exemplified by Factor 1), or they wanted to explore their personal relation to the research (exemplified by Factor 2) or they wanted to seek practical and emotional support to navigate their lived and professional experiences (exemplified by Factor 3) through supervision. This research is a stepping stone towards developing the evidence base for mandatory research supervision integrating components of clinical supervision for all lived experience researchers.

### Electronic supplementary material

Below is the link to the electronic supplementary material.

Supplementary Material 1

## Data Availability

No datasets were generated or analysed during the current study.

## Abbreviations

ARC: Applied Research Collaboration
CQC: Care Quality Commission
The EMERGES: Framework (Empowerment, Motivation to integrate, Empathy of the self and others, Recovery model and medical model, Growth and transformation, Exclusion and Survivor roots
NHS: National Health Service
NIHR: National Institute of Health Research
NSUN: National Survivor User Network
PPI: Patient and Public Involvement
PTSD: Post Traumatic Stress Disorder
SRN: Survivor Researcher Network
UKCGE: UK Council for Graduate Education

## References

[1] Grill C (2021). Involving stakeholders in research priority setting: a scoping review. Res Involv Engagem.

[2] Beames JR, Kikas K, O’Gradey-Lee M, Gale N, Werner-Seidler A, Boydell KM, Hudson JL. A New Normal: integrating lived experience into Scientific Data syntheses. Front Psychiatry. 2021;12. 10.3389/fpsyt.2021.763005. 10.3389/fpsyt.2021.763005 PMC8585932 34777064

[3] National Institute for Health Research (NIHR). PPI (Patient and Public Involvement) resources for applicants to NIHR research programmes. (2019).Https://Www.Nihr.Ac.Uk/Documents/Ppi-Patient-and-Public-Involvement-Resources-for-Applicants-to-Nihr-Research-Programmes/23437#standards-for-Public-Involvement.

[4] Turk A, Boylan A, Locock LA. researcher’s guide to patient and public involvement (2017) https://oxfordbrc.nihr.ac.uk/wp-content/uploads/2017/03/A-Researchers-Guide-to-PPI.pdf.

[5] Sunkel C, Sartor C, Perspectives (2022). Involving persons with lived experience of mental health conditions in service delivery, development and leadership. BJPsych Bull.

[6] National Institute for Health Research (NIHR) Different experiences: A framework for considering who might be involved in research. (2021) https://www.nihr.ac.uk/documents/different-experiences-a-framework-for-considering-who-might-be-involved-in-research/27387.

[7] Faulkner A, Thompson R. Uncovering the emotional labour of involvement and co-production in mental health research. Disabil Soc. 2021;1–24. 10.1080/09687599.2021.1930519.

[8] Hodge S, Competence (2005). Identity and intersubjectivity: applying Habermas’s theory of communicative action to Service user involvement in Mental Health Policy making. Social Theory Health.

[9] Gupta V, Eames C, Golding L (2023). Understanding the identity of lived experience researchers and providers: a conceptual framework and systematic narrative review. Res Involv Engagem.

[10] Cameron C, Moore M, Nutt A, Chambers E (2019). Improving understanding of service-user involvement and identity: collaborative research traversing disability, activism and the academy. Disabil Soc.

[11] Crenshaw K. Demarginalizing the intersection of race and sex: a Black Feminist Critique of Antidiscrimination Doctrine, Feminist Theory and Antiracist politics. University of Chicago Legal Forum; 1989.

[12] Fricker M. *Epistemic Injustice and the Preservation of Ignorance* In: Peels, R. and Blaauw, M, editors The Epistemic Dimensions of Ignorance. Cambridge University Press, pp.144–159. (2016) ISBN 9781107175600.

[13] Munroe W (2016). Testimonial injustice and prescriptive credibility deficits. Can J Philos.

[14] Dodgson JE (2019). Reflexivity in qualitative research. J Hum Lactation.

[15] Olmos-Vega FM, Stalmeijer RE, Varpio L, Kahlke R. A practical guide to reflexivity in qualitative research: AMEE Guide No. 149. *Medical teacher*, 1–11. Advance online publication. (2022). 10.1080/0142159X.2022.2057287. 10.1080/0142159X.2022.2057287 35389310

[16] Jamieson MK, Govaart GH, Pownall M (2023). Reflexivity in quantitative research: a rationale and beginner’s guide. Soc Pers Psychol Compass.

[17] Watharow A, Wayland S. Making qualitative Research Inclusive: Methodological insights in Disability Research. Int J Qualitative Methods. 2022;21. 10.1177/16094069221095316.

[18] Procter & Winter. Personal and relational construct psychotherapy. Palgrave Macmillan. 387 pp. (2020) ISBN 9783030521769.

[19] Lazard & McAvoy (2020). Doing reflexivity in psychological research: what’s the point? What’s the practice?. Qualitative Res Psychol.

[20] Ennis L, Wykes T (2013). Impact of patient involvement in mental health research: longitudinal study. Br J Psychiatry: J Mental Sci.

[21] Proctor B. Training for the supervision alliance attitude, skills and intention. In: Cutliffe JR, Butterworth T, Proctor B, editors. Fundamental themes in Clinical Supervision. Routledge; 2001.

[22] UKCGE Supporting excellent supervisory practice across UKRI doctoral training investments. (2022) https://ukcge.ac.uk/assets/resources/UKRI-Research-Supervision-Report-UKCGE.pdf.

[23] McChesney K. A rationale for trauma-informed postgraduate supervision. Teach High Educ. 2022;1–23. 10.1080/13562517.2022.2145469.

[24] Berger R, Quiros L, Hatzis B. J. The intersection of identities in supervision for trauma-informed practice: Challenges and strategies. Chapter In Trauma-Informed Supervision. First Edition Routledge. (2020) ISBN 9780429330353.

[25] Care Quality Commission (CQC). Regulation 18: Staffing: Health and Social Care Act 2008 (Regulated Activities) Regulations 2014: Regulation 18. (2022).Https://Www.Cqc.Org.Uk/Guidance-Providers/Regulations-Enforcement/Regulation-18-Staffing .

[26] Watts S, Stenner P. Doing Q Methodological Research: theory, Method and Interpretation. SAGE Publications Ltd; 2012. 10.4135/9781446251911.

[27] Gupta V. An Exploration of Emerging Identities in Mental Health, Education and Research. PhD Thesis. University of Liverpool. (2023) 10.17638/03171370.

[28] Shinebourne P, Using (2009). Q method in qualitative research. Int J Qualitative Methods.

[29] Banasick S, KADE (2019). A desktop application for Q methodology. J Open Source Softw.

[30] Brown S. Political subjectivity: applications of Q methodology in political science. Yale University Press; 1980.

[31] Kline P. An easy guide to factor analysis. Routledge; 1994.

[32] Dunlop BJ, Woods B, Lovell J, O’Connell A, Rawcliffe-Foo S, Hinsby K (2022). Sharing lived experiences Framework (SLEF): a framework for mental health practitioners when making disclosure decisions. J Social Work Pract.

[33] Bravata DM, Watts SA, Keefer AL, Madhusudhan DK, Taylor KT, Clark DM, Nelson RS, Cokley KO, Hagg HK (2020). Prevalence, predictors, and treatment of Impostor Syndrome: a systematic review. J Gen Intern Med.

[34] Maxwell V. *How Peer Support Training Is Reducing My Imposter Syndrome*. (2020).Https://Www.Psychologytoday.Com/Gb/Blog/Crazy-Life/202011/How-Peer-Support-Training-Is-Reducing-My-Imposter-Syndrome.

[35] Simpson A, Oster C, Muir-Cochrane E (2018). Liminality in the occupational identity of mental health peer support workers: a qualitative study. Int J Ment Health Nurs.

[36] Kirrane M, Kilroy S, O’Connor C (2019). The moderating effect of team psychological empowerment on the relationship between abusive supervision and engagement. Leadersh Organ Dev J.

[37] Wade JC, Jones JE. Strength-based clinical supervision: a positive psychology approach to clinical training. Springer Publishing Company; 2015.

[38] Poremski D, Kuek JHL, Yuan Q, Li Z, Yow KL, Eu PW, Chua HC (2022). The impact of peer support work on the mental health of peer support specialists. Int J Mental Health Syst.

[39] McPin Foundation. Overcoming imposter syndrome as a peer researcher. (2018). Https://Mcpin.Org/Wsbs-Impostersyndrome/.

[40] Borders LD (1991). A systematic Approach to peer Group Supervision. J Couns Dev.

[41] Lane RC, editor. Psychoanalytic Approaches To Supervision (1st ed.). (1990). Routledge. 10.4324/9781315803708.

[42] Bernard J, Supervisor Training (1979). A discrimination model. Counselor Educ Superv.

[43] Rogers C. *A Theory of Therapy, Personality, and Interpersonal Relationships: As Developed in the Client-Centered Framework* In S. Koch, editor, Psychology: A Study of a Science. Formulations of the Person and the Social Context (Vol. 3, pp. 184–256). (1959). McGraw Hill.

[44] Mental Health Foundation. *Debt and Mental Health*. (2022). Https://Www.Mentalhealth.Org.Uk/Explore-Mental-Health/a-z-Statements/Debt-and-Mental-Health.

[45] Faulkner A. The ethics of survivor research guidelines for the ethical conduct of research carried out by mental health service users and survivors. The Policy; 2004.

[46] Wampold BE (2015). How important are the common factors in psychotherapy? An update. World Psychiatry: Official J World Psychiatric Association (WPA).

[47] Gupta V. The 3 types of lived experience researcher and how to support them. McPin Foundation Blog. (2023) https://mcpin.org/3-types-of-lived-experience-researcher/.

