# Supplementary material for: Identifying the priorities for supervision by lived experience researchers: a Q sort study
Source: Res Involv Engagem. 2024 Jun 25;10:66. doi: 10.1186/s40900-024-00596-w (PMC11197215; doi:10.1186/s40900-024-00596-w)
Supplement: Supplementary file 1 — Supplementary Material 1 [file 40900_2024_596_MOESM1_ESM.docx]

**Supplementary materials**

**Feedback requested from participants**

1. Please explain why you prioritised your top 3 items as most useful to you.
2. Please explain why you sorted your bottom 3 items as least useful to you.
3. Are there any items you had to place lower down/higher up due to the limits of the grid? If so, please tell me about why you feel some statements should be better or less prioritised.
4. Is there anything you would like to explain about the way you sorted the usefulness of these topics for supervision?
5. What other topics of discussion would be useful to you to explore in supervision based on your role as a lived experience researcher?
6. Is there anything else you would like to share?

**Supplementary materials**

**Table 5. Distinguishing statements for Factor 1**

| Distinguishing statements | **F1** | F2 | F3 |
| --- | --- | --- | --- |
| Discussing how to use the skills and abilities I already have and apply them in my role* | **+4** | +2 | -3 |
| Strengthening my identity as a lived experience researcher* | **+4** | -1 | 0 |
| Identifying my training and learning needs relevant to the research* | **+4** | +1 | -1 |
| Enabling me to reflect on what motivates and enables me to do my role* | **+4** | +2 | 0 |
| Helping me to identify the positive experiences I have in my role* | **+3** | -2 | -3 |
| Having conversations in which there is recognition of my growth in my role* | **+3** | -1 | -2 |
| Helping me to increase my confidence in my role as a lived experience researcher* | **+2** | -1 | -1 |
| Managing the expectations that I and others have of me in this role* | **+2** | -4 | +5 |
| *Significant at p <0.01 |  |  |  |

**Table 6. Distinguishing statements for Factor 2**

| Distinguishing statements | F1 | **F2** | F3 |
| --- | --- | --- | --- |
| Providing space to discuss the impact on me when seeing people like me suffer through the data | -3 | **+5** | -1 |
| Providing space for me to talk about my current lived experiences and how this may impact the research* | +3 | **+5** | +1 |
| Regularly discussing and reviewing my wellbeing and support needs* | +2 | **+4** | +1 |
| Exploring how I relate to the participants in the research* | -2 | **+4** | -3 |
| Exploring how I relate to the research topic* | 0 | **+4** | -4 |
| Helping me to reflect on how I feel when assumptions are made of me due to my lived experience* | -3 | **+4** | -1 |
| Helping me to reflect on how I feel when research findings are similar to my own experiences* | -5 | **+2** | 0 |
| Helping me to reflect on how I feel when research findings are different to my own experiences* | -2 | **+2** | -2 |
| Discussing how to use the skills and abilities I already have and apply them in my role* | +4 | **+2** | -3 |
| *Significant at p <0.01 |  |  |  |

**Table 7. Distinguishing statements Factor 3**

| Distinguishing statements | F1 | F2 | **F3** |
| --- | --- | --- | --- |
| Managing the expectations that I and others have of me in this role* | +2 | -4 | **+5** |
| Helping me to reflect on times when I am prevented from making meaningful change* | -1 | 0 | **+4** |
| Supporting me with issues around payment for my work by my employer* | -1 | -5 | **+4** |
| Helping me to reflect on barriers I come across in my role* | 0 | +1 | **+4** |
| Exploring how to navigate lived experience and professional aspects of the work at the same time* | -1 | -2 | **+3** |
| Exploring what it means to be a lived experience researcher and enabling me to better understand the role* | 0 | 0 | **+3** |
| Discussing aspects of myself that are known to others and how this may impact my work* | 0 | 0 | **+3** |
| *Significant at p <0.01 |  |  |  |
